# Supplementary figures and images for: SIV-specific neutralizing antibody induction following selection of a PI3K drive-attenuated nef variant
Source: eLife. 2025 Mar 3;12:RP88849. doi: 10.7554/eLife.88849 (PMC11875539; doi:10.7554/eLife.88849)

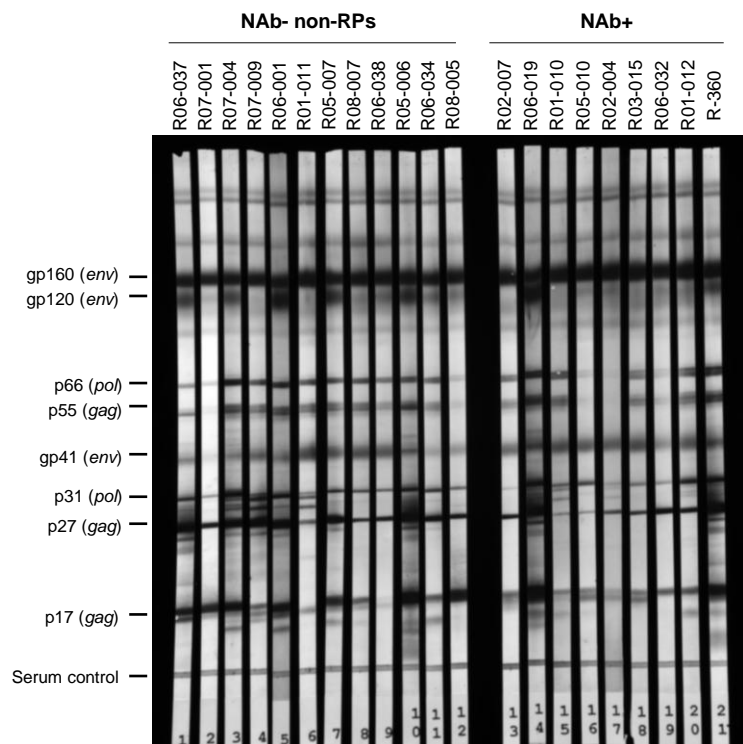

Supplement: Figure 1—figure supplement 3—source data 1. [file elife-88849-fig1-figsupp3-data1.zip › Figure 1—figure supplement 3 source data 1. PDF file containing original western blots for Figure 1—figure supplement 3, labeling the corresponding viral protein bands and IDs/Original western blots for Figure 1—figure supplement 3^J labeling the corresponding viral protein bands and IDs.pdf]

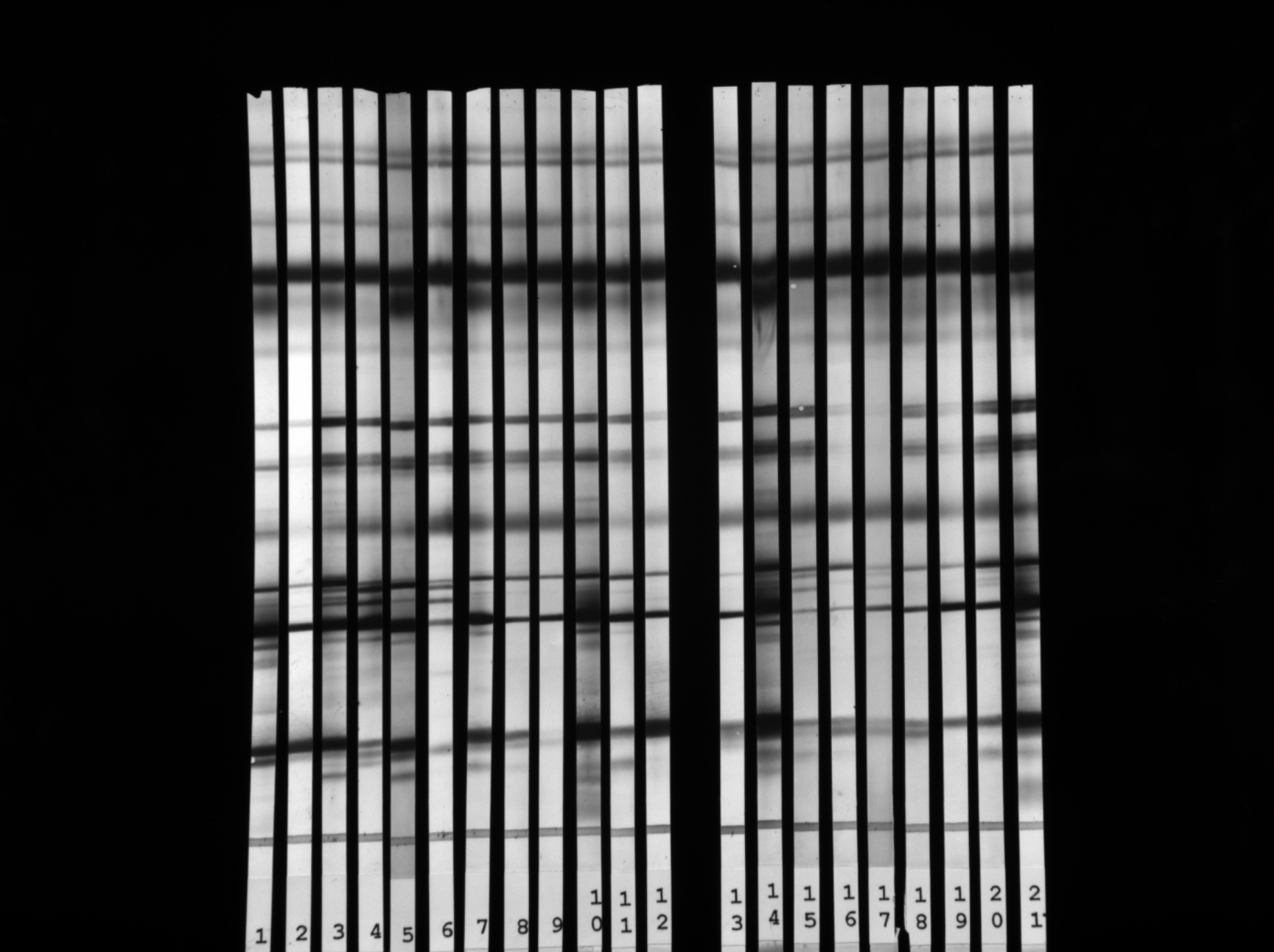

Supplement: Figure 1—figure supplement 3—source data 2. [file elife-88849-fig1-figsupp3-data2.zip › Figure 1—figure supplement 3 source data 1. Original file for western blot analysis displayed in Figure 1—figure supplement 3/BioRad 2014-06-11 01hr 41min.tif]

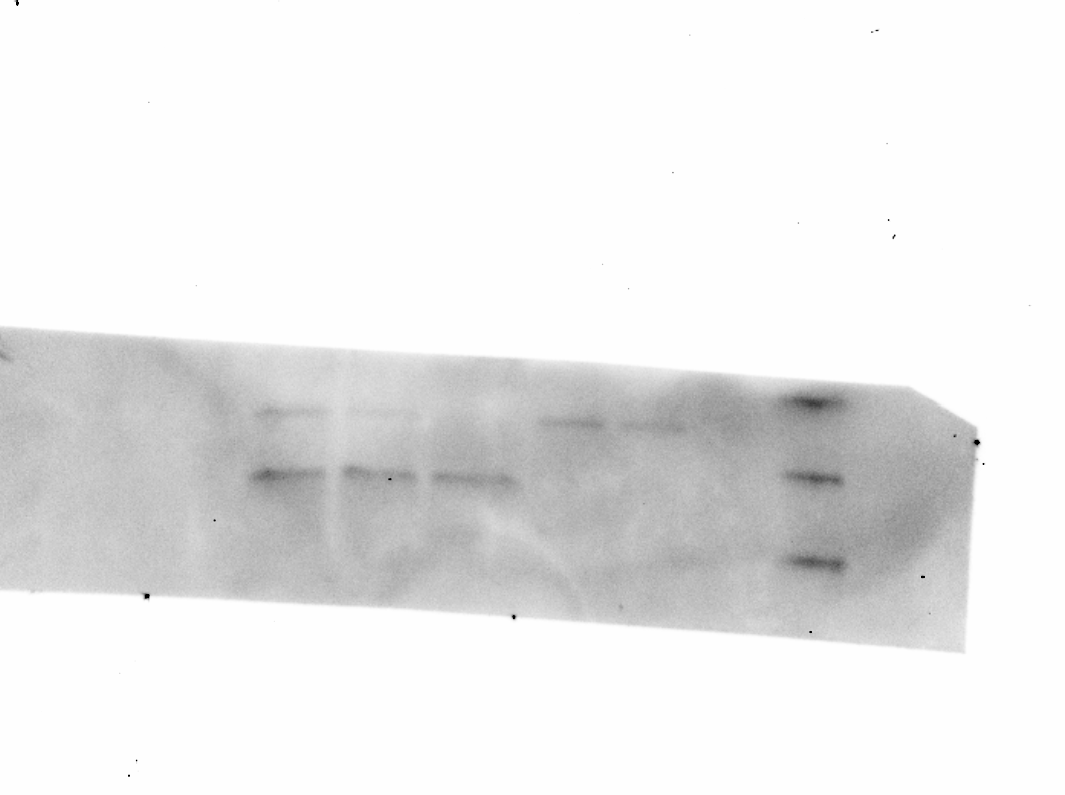

Supplement: Figure 4—source data 2. [file elife-88849-fig4-data2.zip › Figure 4-source data 2. Original files for western blot analysis displayed in Figure 4E/2024-05-21 22hr 26min-MKpre-analysis.png]
