# Supplementary material for: SIV-specific neutralizing antibody induction following selection of a PI3K drive-attenuated nef variant
Source: eLife. 2025 Mar 3;12:RP88849. doi: 10.7554/eLife.88849 (PMC11875539; doi:10.7554/eLife.88849)
Supplement: Figure 4—source data 1. [file elife-88849-fig4-data1.zip › Figure 4-source data 1. PDF file containing original western blots for Figure 4E, indicating the relevant bands and treatments/2024-05-21 22hr 26min-MK3 (lane definition + values).pdf]

Image Report: 2024-05-21 22hr 26min-MK3

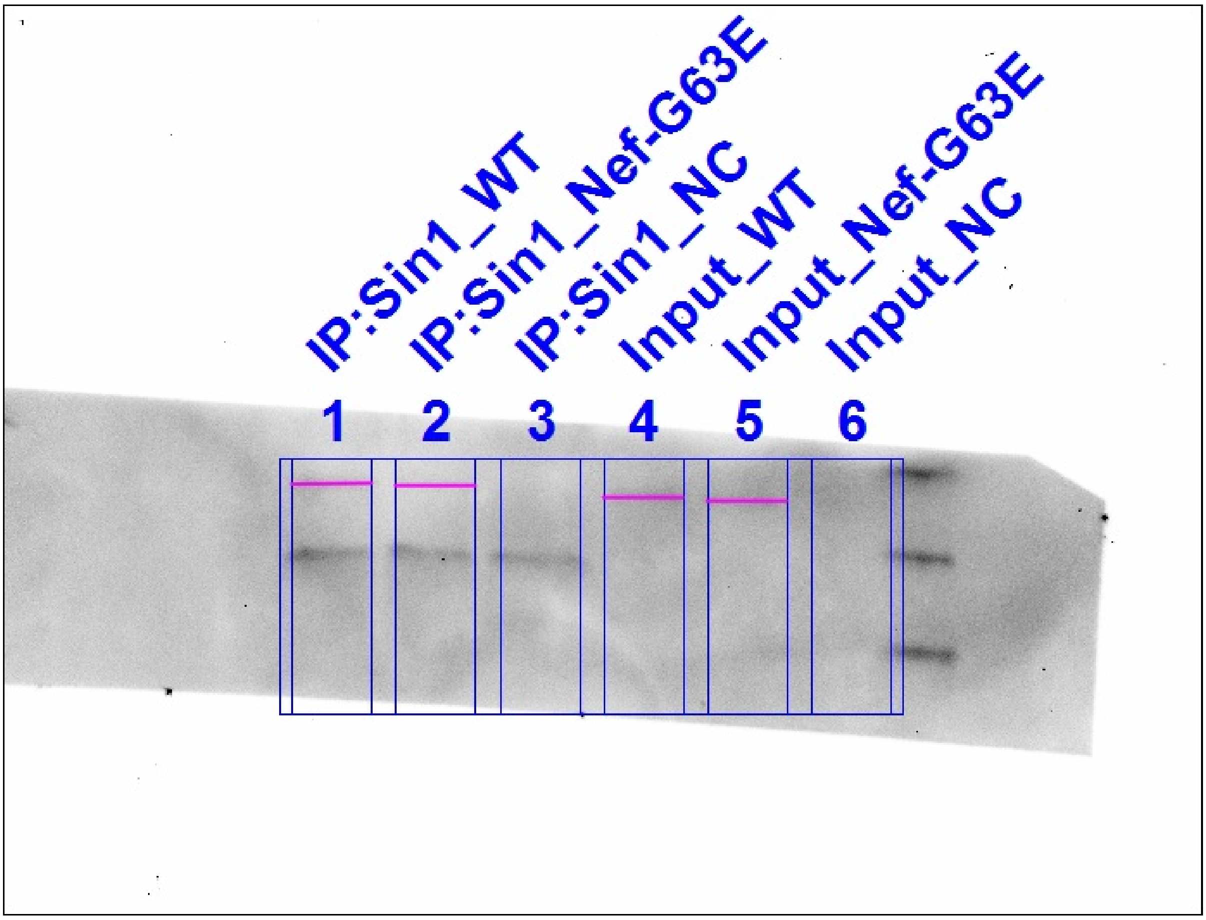

C:/Users/Ken/Desktop/sin/2024-05-21 22hr 26min-MK3.scn

Acquisition Information

Imager	ChemiDoc™ XRS+
Exposure Time (sec)	180.000 (Manual)
Flat Field	Applied (Lens)
Serial Number	721BR04915
Software Version	3.0
Application	Chemi
Excitation Source	No Illumination
Emission Filter	No Filter
Binning	2x2

Image Information

Acquisition Date	5/21/2024 10:29:23 PM
User Name	Ken
Image Area (mm)	X: 90.0 Y: 67.2
Image Pixels	X: 696 Y: 520
Pixel Size (um)	X: 129.3 Y: 129.3
Data Range (Int)	0 - 45380

Analysis Settings

Detection	Lane detection:<br>Manually created lanes<br><br>Band detection:<br>Automatically detected bands with sensitivity: High<br>Manually adjusted bands

	Lane Background Subtraction:<br>Lane background subtracted with disk size: 10

Lane And Band Analysis

Lane 1 - IP:Sin1_WT

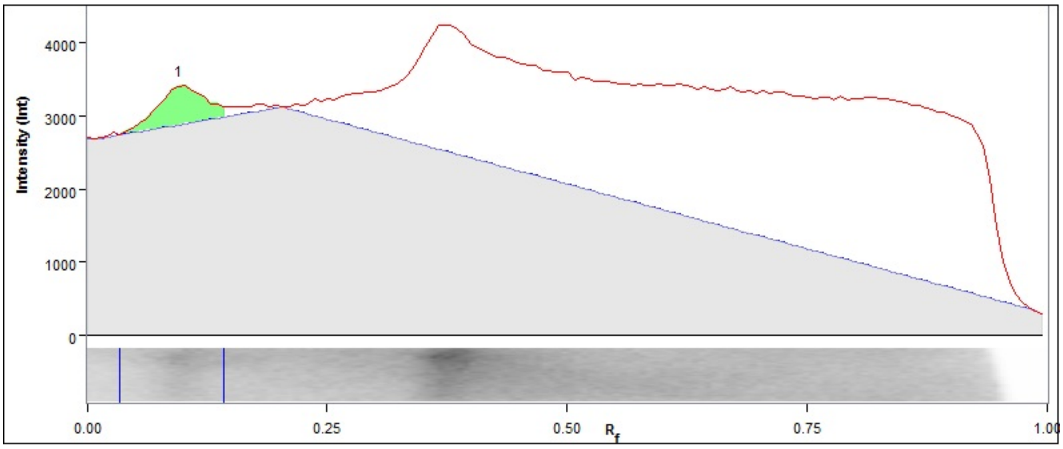

Band No.	Band Label	Mol. Wt. (KDa)	Relative Front	Volume (Int)	Abs. Quant.	Rel. Quant.	Band %	Lane %
1		N/A	0.101	212,934	N/A	N/A	100.0	2.5

Band Detection	Automatically detected bands with sensitivity: High
Lane Background	Lane background subtracted with disk size: 10

Lane 2 - IP:Sin1_Nef-G63E

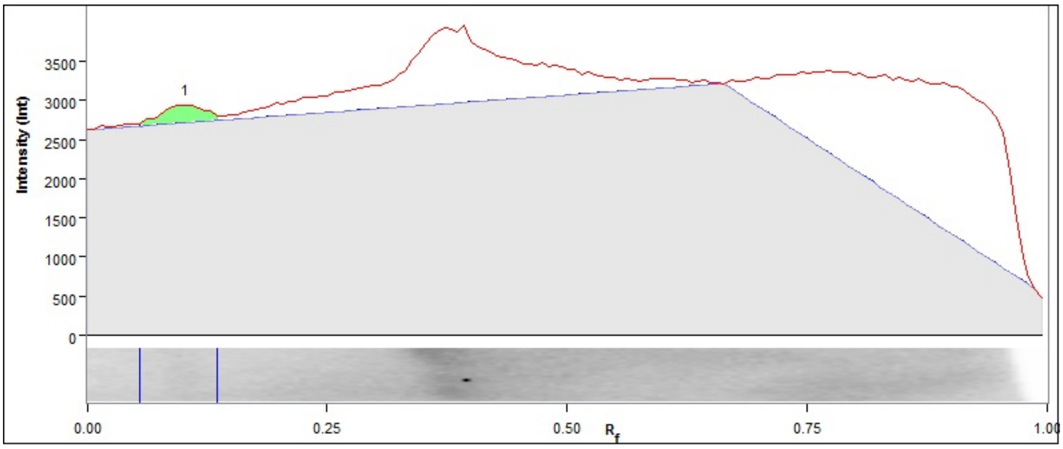

Band No.	Band Label	Mol. Wt. (KDa)	Relative Front	Volume (Int)	Abs. Quant.	Rel. Quant.	Band %	Lane %
1		N/A	0.108	84,410	N/A	N/A	100.0	2.2

Band Detection	Automatically detected bands with sensitivity: High
Lane Background	Lane background subtracted with disk size: 10

Lane 3 - IP:Sin1_NC

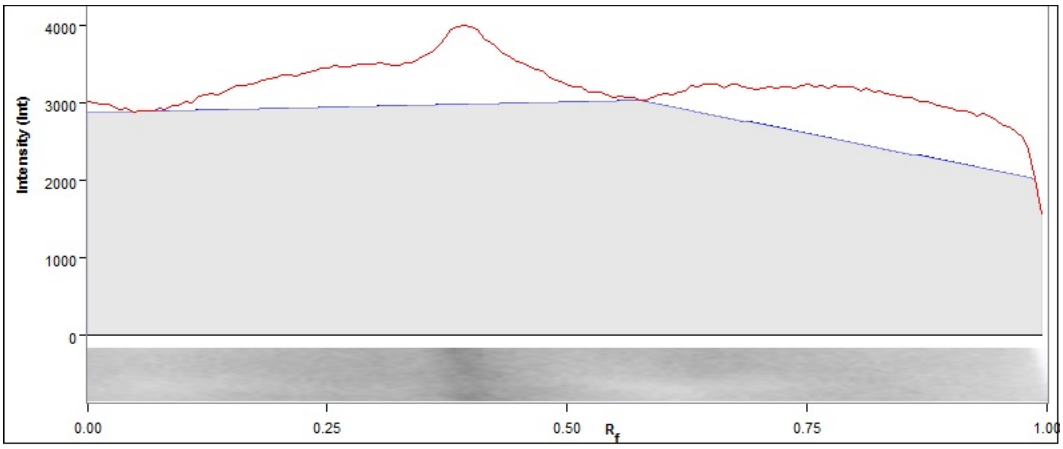

Band No.	Band Label	Mol. Wt. (KDa)	Relative Front	Volume (Int)	Abs. Quant.	Rel. Quant.	Band %	Lane %

Band Detection	Automatically detected bands with sensitivity: High
Lane Background	Lane background subtracted with disk size: 10

Lane 4 - Input_WT

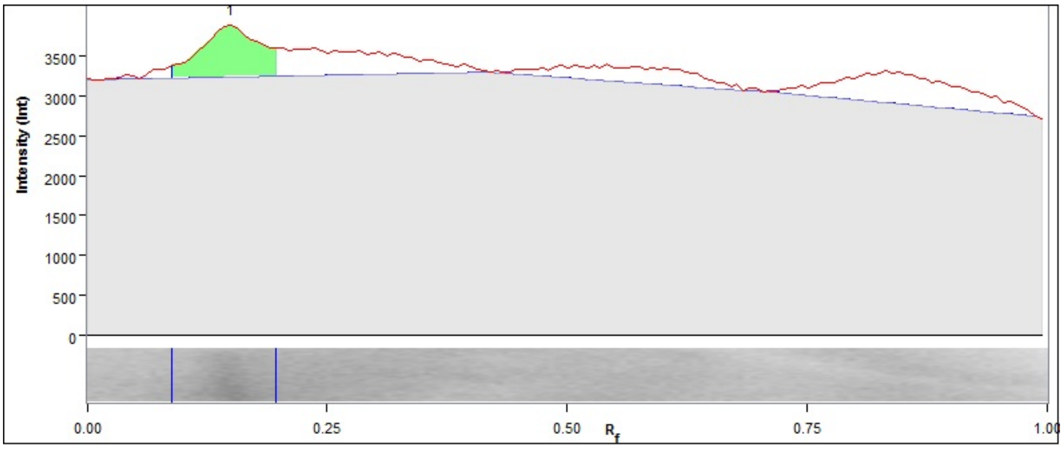

Band No.	Band Label	Mol. Wt. (KDa)	Relative Front	Volume (Int)	Abs. Quant.	Rel. Quant.	Band %	Lane %
1		N/A	0.155	318,504	N/A	N/A	100.0	23.6

Band Detection	Automatically detected bands with sensitivity: High
Lane Background	Lane background subtracted with disk size: 10

Lane 5 - Input_Nef-G63E

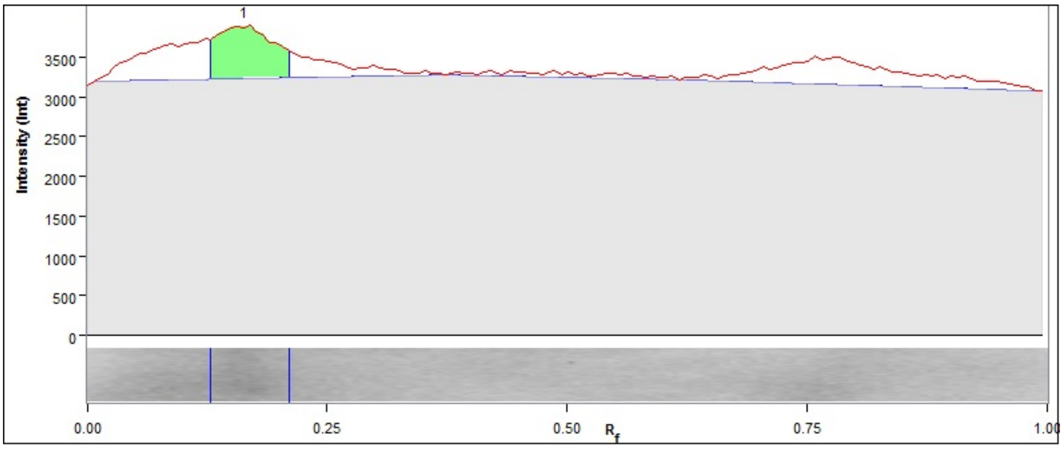

Band No.	Band Label	Mol. Wt. (KDa)	Relative Front	Volume (Int)	Abs. Quant.	Rel. Quant.	Band %	Lane %
1		N/A	0.169	339,434	N/A	N/A	100.0	28.3

Band Detection	Automatically detected bands with sensitivity: High
Lane Background	Lane background subtracted with disk size: 10

Lane 6 - Input_NC

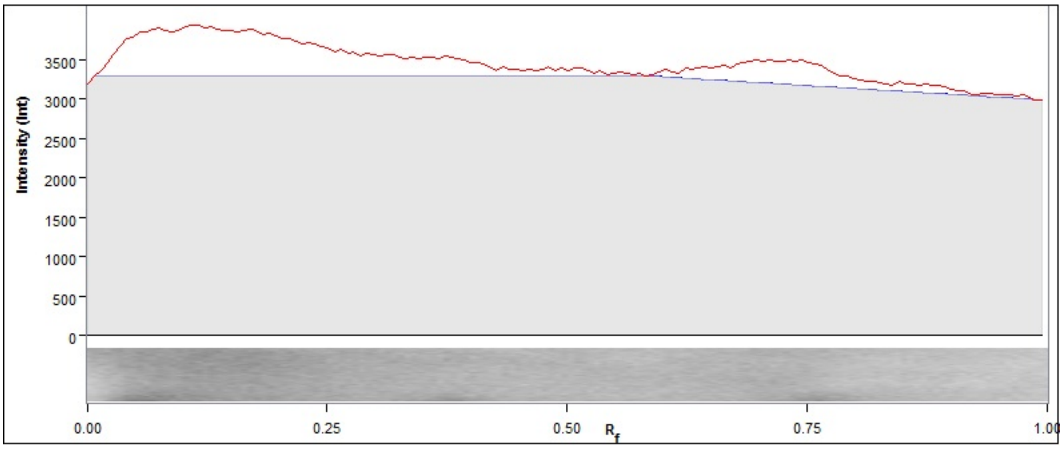

Band No.	Band Label	Mol. Wt. (KDa)	Relative Front	Volume (Int)	Abs. Quant.	Rel. Quant.	Band %	Lane %

Band Detection	Automatically detected bands with sensitivity: High
Lane Background	Lane background subtracted with disk size: 10
